# Cloning and Characterization of Two Toll Receptors (*PcToll5* and *PcToll6*) in Response to White Spot Syndrome Virus in the Red Swamp Crayfish *Procambarus clarkii*

**DOI:** 10.3389/fphys.2018.00936

**Published:** 2018-07-16

**Authors:** Ying Huang, Yihong Chen, Kaimin Hui, Qian Ren

**Affiliations:** ^1^College of Marine Science and Engineering, Nanjing Normal University, Nanjing, China; ^2^College of Oceanography, Hohai University, Nanjing, China; ^3^Co-Innovation Center for Marine Bio-Industry Technology of Jiangsu Province, Lianyungang, China; ^4^Guangdong Provincial Key Laboratory of Marine Resources and Coastal Engineering, South China Sea Bio-Resource Exploitation and Protection Collaborative Innovation Center, School of Marine Sciences, Sun Yat-sen University, Guangzhou, China

**Keywords:** *Procambarus clarkii*, WSSV, Toll receptors, innate immunity, over-expression, RNAi

## Abstract

Toll/Toll-like receptors are key components in the innate immune responses of invertebrates. In this study, we identified two novel Toll receptors (*PcToll5* and *PcToll6*) from the red swamp crayfish *Procambarus clarkii*. The complete cDNA sequence of *PcToll5* is 4247 bp, encoding a 1293 amino acid polypeptide. The full-length 4688 bp *PcToll6* encodes a putative protein of 1195 amino acids. Quantitative RT-PCR analysis indicated that *PcToll5* and *PcToll6* were constitutively expressed in all tissues studied. The highest expression levels of *PcToll5* and *PcToll6* were found in the intestine and gills, respectively, and were significantly upregulated from 24 to 48 h during white spot syndrome virus (WSSV) challenge. siRNA-mediated RNA interference results showed that *PcToll5* and *PcToll6* might regulate the expression of anti-lipopolysaccharide factors (*PcALF2* and *PcALF3*) *in vivo*. Overexpression of PcToll5 and PcToll6 in *Drosophila* Schneider 2 (S2) cells activated the transcription of *Drosophila* antimicrobial peptides, including *drosomycin* (*Drs*), *metchnikowin* (*Mtk*), and *attacin A* (*AttA*), and shrimp *Penaeidin*-4 (*Pen4*). These findings provide significant information that *PcToll5* and *PcToll6* may contribute to host immune defense against WSSV in *P. clarkii*.

## Introduction

The red swamp crayfish *Procambarus clarkii* is one of the most important farmed freshwater crayfish species worldwide. However, the sustainable culture of crayfish in China has been hindered by disease outbreaks caused by white spot syndrome virus (WSSV) (Maeda et al., 2000). White spot syndrome (WSS) caused by WSSV is the common destructive virus-induced disease (Arts et al., 2007). Similar to other invertebrates, giant freshwater prawns lack an acquired immune system and relies mainly on their innate immunity to fight against invading foreign microbes (Medzhitov and Janeway, 2000a,b). Therefore, the innate immunity of this species in response to pathogen invasion should be studied (Buchmann, 2014).

The innate immune system constitutes the first barrier of defense against pathogen invasion in crustaceans and has been conserved throughout the evolutionary process. Upon infection, host germ line-encoded pattern-recognition receptors (PRRs) identify and bind to pathogen-associated molecular patterns (PAMPs) located on the surfaces of microorganisms to trigger multiple downstream signaling pathways (Janeway and Medzhitov, 2002; Medzhitov and Janeway, 2002; Huang et al., 2013). Scholars have studied several PRRs, such as Toll or Toll-like receptors (TLRs), RIG-like receptors, NOD-like receptors, and C-type lectins (Christophides et al., 2004; Huang et al., 2018). Diverse PRRs react with specific PAMPs, exhibit different expression patterns, activate specific signal pathways, and lead to different anti-pathogen responses (Akira et al., 2006).

As one of the most widely studied PRRs, TLRs are involved in pathogen recognition and activation of immune responses (Uematsu and Akira, 2006; Medzhitov, 2007). The first Toll from *Drosophila melanogaster* is a necessary gene product for embryonic dorsoventral polarity development (Leulier and Lemaitre, 2008; Valanne et al., 2011). This Toll also sense microbial pathogens, such as mammalian TLRs (Lemaitre et al., 1996; Akira et al., 2006). To date, studies have identified 1 Toll-like protein in *Caenorhabditis elegans* (Tenor and Aballay, 2008), 9 Toll proteins in *Drosophila* (Valanne et al., 2011), and 10–12 TLRs in mammals (Roach et al., 2005; Akira et al., 2006). A large number of Toll proteins from crustaceans have been widely investigated. In shrimp, Tolls have been studied in *Fenneropenaeus chinensis*, *Penaeus monodon*, *Marsupenaeus japonicus*, *Litopenaeus vannamei*, and *P. clarkii* (Arts et al., 2007; Yang et al., 2008; Mekata et al., 2008; Wang et al., 2012, 2015). In the giant tiger shrimp *P. monodon*, a Toll homolog was expressed in the intestine, gills, and hepatopancreas and could be involved in defense against pathogens (Arts et al., 2007). A Toll receptor from the Chinese shrimp *F. chinensis* was also expressed in different tissues; the expression was the highest in the lymphoid organ and regulated after *Vibrio anguillarum* or WSSV stimulation (Yang et al., 2008). A new type of Toll receptor gene was found to be expressed by 76-fold higher than that the control stimulated with peptidoglycan at 12 h in the lymphoid organ of the kuruma shrimp *Marsupenaeus japonicus* (Mekata et al., 2008). Three different Tolls (Toll1–3) have been studied in the white leg shrimp *L. vannamei* based on their protein similarities; all these Tolls met the challenges with *Vibrio alginolyticus* or WSSV (Wang et al., 2012). In the freshwater crayfish *P. clarkii*, *PcToll* was upregulated in different tissues after challenge with *V. anguillarum* or *Staphylococcus aureus* and was found to be involved in regulation of the expression of antimicrobial peptides (AMPs), including *crustins* (*Cru1* and *Cru2*), *anti-lipopolysaccharide factor* 2 (*ALF2*), and *lysozyme* 1 (*Lys1*) (Wang et al., 2015). Silencing *P. clarkii* Toll (*PcToll3*) influenced the expression of myeloid differentiation factor 88 (*PcMyd88*), tumor necrosis factor-associated factor 6 (*PcTRAF6*), and *PcDorsal*, which were the counterparts of the *Drosophila* Toll signaling pathway (Lan et al., 2016). In crabs, Toll reporters were identified in three crab species, one in *Scylla paramamosain* (Lin et al., 2012), two in *Eriocheir sinensis* (Yu et al., 2013), and three in *Portunus trituberculatus* (Zhou et al., 2015); these reporters were responsive to bacterial pathogens or PAMPs.

In this study, the complete cDNA sequences of two novel Tolls (*PcToll5* and *PcToll6*) from *P. clarkii* were identified and their responses to WSSV challenge were investigated. This research will be potentially helpful in understanding the innate immune defense of economically important crayfish.

## Materials and Methods

### WSSV Challenge and Tissue Collection

One hundred healthy red swamp crayfish (about 15 g each) were obtained from an agricultural market in Nanjing (Jiangsu Province, China). They were acclimated in fresh water in laboratory tanks at 25°C for a week before the experiments. Hemolymph was collected from at least five crayfish, mixed with 1/3 volume of anticoagulant buffer (10% sodium citrate, pH 7.0) containing 200 mM phenylthiourea, and centrifuged at 800 *g* at 4°C for 10 min to isolate hemocytes. Five tissues including heart, hepatopancreas, gill, stomach, and intestine were quickly collected. For the viral challenge experiments, crayfish were divided into two groups (20 crayfish in each group). Each crayfish in group 1 was challenged with 100 μl of WSSV (10^5^ copies/mL). Each crayfish in group 2 was injected with 100 μl of PBS and used as blank control (Huang Y. et al., 2016). At 0, 24, 36, and 48 h post injection (hpi), the intestines were randomly extracted from five crayfish of each group.

### Total RNA Extraction and First-Strand cDNA Synthesis

Total RNA samples from different tissues and the intestine of WSSV-challenged crayfish were extracted in accordance with the manufacturer’s instructions (Spin-column, BioTeke, Beijing, China). The 5′ and 3′ cDNA sequences for the rapid amplification of cDNA ends (RACE) were synthesized using the intestine total RNA as template to obtain the full lengths of *PcToll5* and *PcToll6* genes. First-strand cDNA (5′ cDNA and 3′ cDNA) was synthesized using 5′-CDS primer A (5′-T_25_VN-3′), SMARTer IIA oligo (5′-AAGCAGTGGTAT CAACGCAGAGTACXXXXX-3′), 3′-CDS primer [5′-AAGCA GTGGTATCAACGCAGAGTAC(T)30VN-3′], and a Clontech SMARTer^TM^ RACE cDNA Amplification kit from Takara (Dalian, China). First-strand cDNA synthesis of different samples for qRT-PCR analysis was performed using the PrimeScript^®^ First-strand cDNA Synthesis Kit (Takara, Dalian, China) with the Oligo dT Primer (Ren et al., 2014).

### cDNA Cloning of *PcToll5* and *PcToll6*

Two expressed sequence tags (ESTs) in *P. clarkii* similar to TLR genes were obtained from our previous high-throughput transcriptome data. On the basis of the original EST sequences, the following gene-specific primers were designed to clone the full-length cDNAs of *PcToll5* and *PcToll6*, respectively: (PcToll5-F: 5′-CGCCTGTGAGGTGTGACCACTATGT-3′, PcToll5-R: 5′-ACATCCAGAACCACCAGGCGAATAAGC-3′; and PcToll6-F: 5′-GTGTCGTTTTGAGTTCCGTTCCGCCC-3′, PcToll6-R: 5′-ACCAATCGGTGTTGTAGGTCCGCAGC-3′). The Advantage 2 PCR Kit from Takara (Dalian, China) was used for gene cloning. The PCR amplification conditions were as follows: five cycles at 94°C for 30 s and 72°C for 3 min; five cycles at 94°C for 30 s, 70°C for 30 s, and 72°C for 3 min; and 20 cycles at 94°C for 30 s, 68°C for 30 s, and 72°C for 3 min (Huang Y. et al., 2016). The PCR products were cloned into the pEASY^®^-T1 vector (TransGen Biotech) and sequenced (Invitrogen).

### Bioinformatics Analysis

Online BLAST program^1^ was used to search the homology comparisons of nucleotide and amino acid sequences. cDNA translation, pI analysis, and molecular mass prediction were performed by ExPASy.^2^ Domain and signal peptide predictions were conducted by SMART^3^. Multiple protein sequence alignment was performed using MEGA 5.05 and analyzed on GENEDOC software. MEGA 5.05 was utilized to produce phylogenetic trees, and NJ (Neighbor-Joining) method was applied to phylogenetic analysis (Kumar et al., 2008).

### Quantitative Real-Time PCR (qRT-PCR)

Tissue distribution and expression profiles of *PcToll5* and *PcToll6* were analyzed by qRT-PCR with the prepared cDNA. *PcToll5* and *PcToll6* were amplified with specific primers (PcToll5-RT-F: 5′-GCAAACCGCATCAAAGCGACCA-3′ and PcToll5-RT-R: 5′-GCAGCAGCAAGCAGCAGCAACA-3′; PcToll6-RT-F: 5′-GCCTTCGCTGTTCTCCTCACC-3′ and PcToll6-RT-R: 5′-TCCCC TTCATACTCGCTCCTG-3′), respectively. The procedure of qRT-PCR consisted of the following: 95°C for 30 s, followed by 40 cycles of 95°C for 5 s, and 60°C for 30 s. Melting curve analysis was conducted to confirm the specificity of qRT-PCR amplification. Glyceraldehyde 3-phosphate dehydrogenase (GAPDH) was also amplified for internal standardization with the corresponding primers (PcGAPDH-RT-F: 5′-CAATGTTC GTCTGTGGAGTGA-3′ and PcGAPDH-RT-R: 5′-GGAAGAT GGGATGATGTTCTG-3′). Comparative threshold cycle method (2^-ΔΔCT^) was used to calculate the transcript expression levels (Livak and Schmittgen, 2001). The experiment was repeated three times.

### Dual Luciferase Activity Assay in S2 Cells

The expression vector for the full-length PcToll5 or PcToll6 was constructed using pAc5.1/V5-His B (Invitrogen, United States). The PCR products were amplified with the specific primers (PcToll5-pAc-F: 5′-CCCGGATCGGGGTACCATGCTCAGCCG CTTGGAGGCCCTTG-3′ and PcToll5-pAc-R: 5′-TTCGAACCG CGGGCCCTCTAAAGACGGCATTGCTCTGCGT-3′; PcToll6-pAc-F: 5′-CCCGGATCGGGGTACCATGCTCCAGGATGTGAC AGTTCTG-3′, and PcToll6-pAc-R: 5′-TTCGAACCGCGGGC CCTCAGTGTCGCCCCATTTAAGATAGG-3′). After digestion with *Kpn* I and *Apa* I (Takara, Dalian, China), the PCR products were cloned into pAc5.1/V5-His B. The positive clones were sequenced to ensure correct insertion. On account of the unavailability of a crayfish cell line, *Drosophila* Schneider 2 (S2) cells (Invitrogen, United States) were used to explore the activation of AMP transcription of PcToll5 and PcToll6. S2 cells were cultured in *Drosophila* serum-free medium SDM (Invitrogen, United States) augmented with 10% fetal bovine serum (Invitrogen) at 27°C. The cells were seeded overnight for DNA transfection, and the plasmids were transfected using the Cellfectin II reagent (Invitrogen). Dual-luciferase reporter assays were carried out by transfecting S2 cells in 96-well plates (TPP, Switzerland) by using 0.3 g of expression plasmids, 0.2 g of reporter gene plasmids, and 0.02 g of pRL-TK renilla luciferase plasmid (Promega, United States) as control. The reporter gene plasmids were constructed using the promoter sequences of the following genes: *L. vannamei* AMP *penaeidin*-4 (*Pen4*), *Drosophila* AMPs, *metchnikowin* (*Mtk*), *drosomycin* (*Drs*), and *attacin A* (*AttA*). All the assays were performed in triplicate. At 48 h post-transfection, firefly and renilla luciferase activities were measured on the Dual-Luciferase Reporter Assay System (Promega, United States) (Huang et al., 2015).

### siRNA-Mediated RNA Interference Assay and Expression Patterns of *ALFs*

Based on the sequences of *PcToll5* and *PcToll6* genes, siRNAs specifically targeting *PcToll5* and *PcToll6* were synthesized *in vitro* by using a commercial kit (Takara, Japan) according to the manufacturer’s instructions. The siRNAs used were as follows: PcToll5-siRNA (5′-GCACCGAAACAUUCUCAUA-3′) and PcToll6-siRNA (5′-GCUCUUCUUUCAAAGUGCACUUCAA-3′). The sequences of the siRNAs were scrambled to generate the following: control PcToll5-siRNA-scrambled (5′-ACGCAA GACAUCUAUACCU-3′) and PcToll6-siRNA-scrambled (5′-GU CUUACUCUCAUGCAUAGUUCACA-3′). The synthesized siRNAs were dissolved in siRNA buffer and quantified by spectrophotometry (Huang et al., 2014).

Healthy crayfish were assigned to five groups. Crayfish in group 1 or 2 were injected with 20 μg of PcToll5-siRNA or PcToll6-siRNA, respectively. At 16 h after the injection, PcToll5-siRNA or PcToll6-siRNA (20 μg) with WSSV (10^5^ copies/mL) (100 μl/crayfish) were injected into the same crayfish. Crayfish in groups 3 or 4 were initially injected with 20 μg of PcToll5-siRNA-scrambled or PcToll6-siRNA-scrambled, respectively. After 16 h, 20 μg of PcToll5-siRNA-scrambled or PcToll6-siRNA-scrambled and WSSV was injected into the same crayfish. At 36 h after the last injection, the intestine was isolated from five crayfish for RNA extraction and first-strand cDNA synthesis. qRT-PCR analysis was conducted to determine the efficiency of RNAi. After RNAi assay, the expression levels of ALF genes were determined through qRT-PCR analysis. The gene expression levels of *PcALF2* and *PcALF3* were analyzed in the intestine. The primers used were PcALF2-RT-F: 5′-CGTGGGAGTGTTTGTGGTGGT-3′ and PcALF2-RT-R: 5′-TTGGACTGTAACTGTAGCGGC-3′; PcALF3-RT-F: 5′-AGGTGTTGAAGATGAAGTGGT-3′ and PcALF3-RT-R: 5′-GCTTGTTGATAATGAGGGTGA-3′.

## Results

### Identification of *PcToll5* and *PcToll6* in *P. clarkii*

**FIGURE 1:**
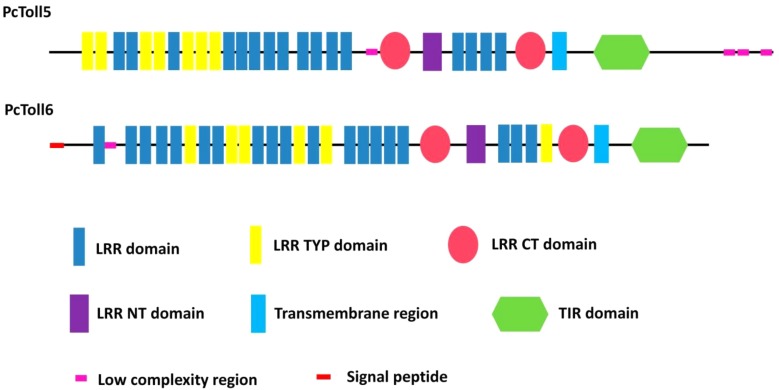
Schematic representation of the domain topology of PcToll5 and PcToll6.

The full length of *PcToll5* (KU680804) is 4247 bp, which includes a 5′ untranslated region (UTR) of 159 bp, a 3882 bp open reading frame (ORF) encoding a protein of 1293 amino acids (aa), and a 3′ untranslated region of 206 bp (Supplementary Figure S1A). The PcToll5 protein contains of 16 leucine-rich repeat (LRR) domains with an aa length of 16–26, 7 LRR domains of 24 aa (belonging to the most populated subfamily LRR TYP), 2 LRR C-terminal (LRR CT) domains of 60 and 53 aa, an LRR N-terminal domain (LRR NT) domain of 38 aa, a transmembrane domain of 23 aa, and an intracellular Toll/interleukin-1 (IL-1) receptor (TIR) domain of 140 aa (**Figure 1**). The molecular weight (MW) and predicted isoelectric point (pI) of PcToll5 are 143.2 kDa and 6.43, respectively. The complete cDNA sequence of *PcToll6* (KU680807) is 4688 bp, which includes a 308 bp 5′ UTR, a 3588 bp ORF encoding an 1195 amino acid polypeptide, and a 792 bp 3′UTR (Supplementary Figure S1B). The PcToll6 protein contains a signal peptide of 18 aa, 19 LRR domains with an aa length of 20 to 30, 6 LRR TYP domains of 24 aa, 2 LRR CT domains of 57 and 53 aa, an LRR NT domain of 39 aa, a transmembrane domain of 23 aa, and a TIR domain of 138 aa (**Figure 1**). PcToll6 has a MW of 136.4 kDa and a pI of 5.70.

### Sequence Analysis of PcToll5 and PcToll6

**FIGURE 2:**
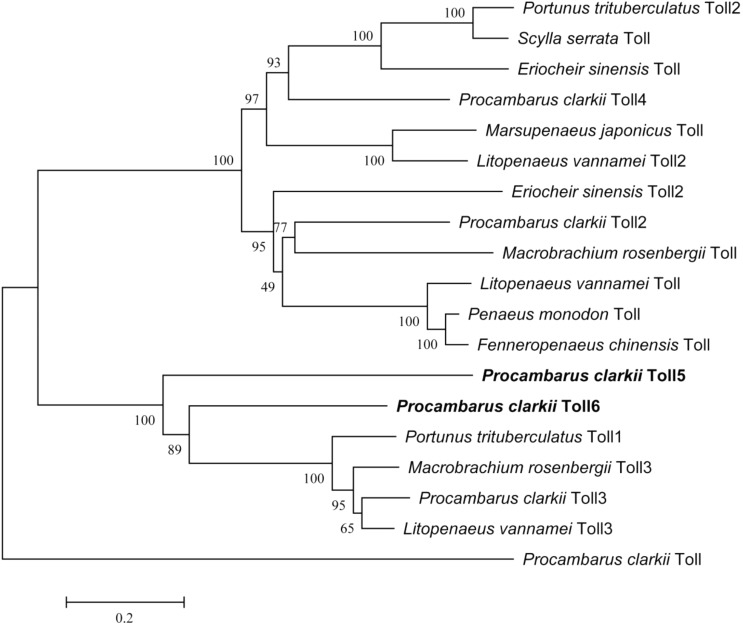
Phylogenetic analysis of PcToll5, PcToll6 and other crustacean Toll proteins. *Eriocheir sinensis* Toll, Toll2: Accession No. AGK90305.1, AGT21374.1; *Fenneropenaeus chinensis* Toll: Accession No. ABQ59330.1; *L. vannamei* Toll, Toll2, Toll3: Accession No. ABK58729.1, AEK86516.1, AEK86517.1; *Macrobrachium rosenbergii* Toll, Toll3: Accession No. AEI25533.1, AHL39102.1; *Marsupenaeus japonicus* Toll: Accession No. BAF99007.1; *Penaeus monodon* Toll: Accession No. ADK55066.1; *Portunus trituberculatus* Toll1, Toll2: Accession No. AKV62617.1, AIZ66853.1; *Scylla serrata* Toll: Accession No. AGG55849.1.

Multiple alignments at the amino acid level showed that PcToll5 and PcToll6 were highly conserved with each other (Supplementary Figure S2). BLASTX result showed that PcToll5 shared 53% identity with Toll from *Parhyale hawaiensis* and 41% identity with Tolls from *Pediculus humanus corporis*, *Athalia rosae*, *Cephus cinctus*, and *Fopius arisanus*. PcToll6 shared 87% identity with Toll from *P. trituberculatus*, 67% identity with Toll from *P. hawaiensis*, and 54% identity with Tolls from *P. humanus corporis*, *Nilaparvata lugens*, *Tribolium castaneum*, and *Zootermopsis nevadensis*. Phylogenetic analysis of the reported crustacean Toll proteins demonstrated that PcToll5 and PcToll6 were clustered together with *P. trituberculatus* Toll1, *Macrobrachium rosenbergii* Toll3, *P. clarkii* Toll3, and *L. vannamei* Toll3 (**Figure 2**).

### Tissue Distribution and Expression Profiles of *PcToll5* and *PcToll6*

**FIGURE 3:**
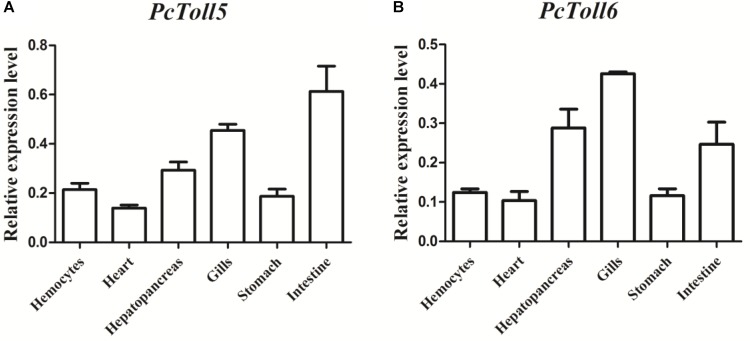
qRT-PCR analysis of *PcToll5* **(A)**, and *PcToll6* **(B)** in the hemocytes, heart, hepatopancreas, gills, stomach and intestine of healthy crayfish. GAPDH was used as an internal control.

**FIGURE 4:**
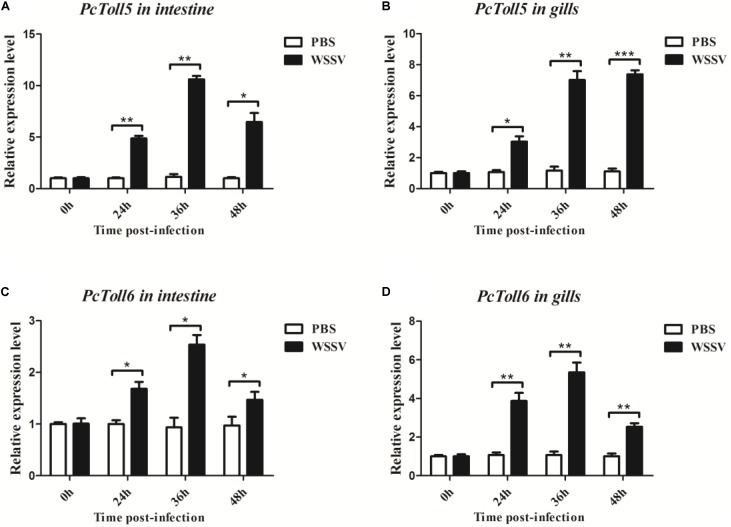
Analysis of *PcToll5* **(A,B)** and *PcToll6* **(C,D)** expression in intestine and gills from crayfish challenged with WSSV using qRT-PCR methods. GAPDH was used as an internal control. Asterisks indicate significant differences (^∗∗∗^*P* < 0.001, ^∗∗^*P* < 0.01, ^∗^*P* < 0.5) compared with values of the control.

*PcToll5* and *PcToll6* were expressed in all tissues studied. The highest expression levels of *PcToll5* and *PcToll6* were found in the intestine and gills, respectively (**Figure 3**). The expression profiles of *PcToll5* and *PcToll6* in the intestine or gills were further examined after WSSV challenge. *PcToll5* and *PcToll6* cDNAs were significantly upregulated in different tissues after challenge with WSSV but showed no significant change in their expression in the PBS-treated group (**Figure 4**). The highest expression of *PcToll5* after challenge with WSSV in the intestine and gills was found at 36 and 48 h post injection, respectively. The *PcToll6* expression reached the highest level at 36 h in the intestine and gills. Hence, *PcToll5* and *PcToll6* might be involved in anti-WSSV immune responses.

### Overexpression of PcToll5 and PcToll6 in *Drosophila* S2 Cells

**FIGURE 5:**
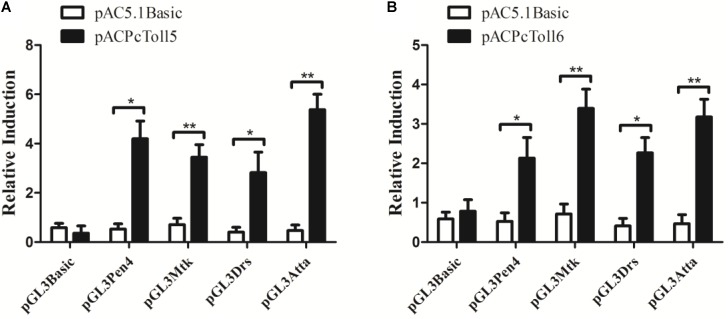
Activation of shrimp (*Pen4*) and *Drosophila* (*Mtk*, *Drs*, *AttA*) AMPs by over-expression of PcToll5 **(A)** or PcToll6 **(B)** in *Drosophila* S2 cells. Significant statistical differences (^∗∗^*P* < 0.01, ^∗^*P* < 0.5) are indicated by asterisks. All data are representative of three independent experiments. The bars indicate the mean 6 SD of the luciferase activity (*n* = 3).

PcToll5 and PcToll6 significantly activated the promoters of *Drosophila* or *L. vannamei* AMP genes (**Figure 5**). PcToll5 induced the promoter activities of AMP *Pen4* by 4.2-fold, *Mtk* by 3.4-fold, *Drs* by 2.8-fold, and *AttA* by 5.4-fold. PcToll6 induced the promoter activities of AMP *Pen4* by 2.1-fold, *Mtk* by 3.4-fold, *Drs* by 2.3-fold, and *AttA* by 3.3-fold.

### *PcToll5* and *PcToll6* Affect the Transcription of *ALFs*

**FIGURE 6:**
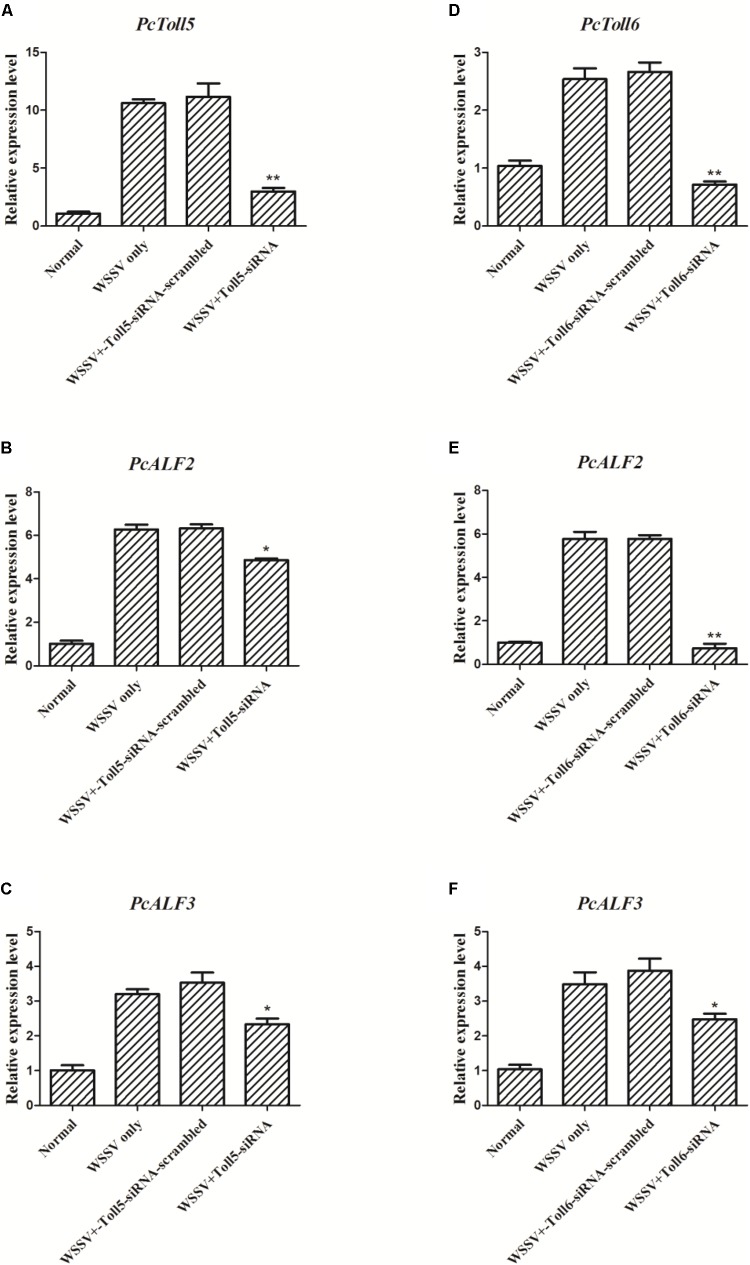
Expression analysis of *PcALF2* and *PcALF3* at 36 h WSSV challenge in intestine of *P. clarkii* (*PcToll5* or *PcToll6* knockdown). qRT-PCR analysis of the RNA interference efficiency of *PcToll5* **(A)** or *PcToll6* **(D)** in *P. clarkii*. The experiments were divided into 4 groups (normal group, 36 h WSSV challenge group, WSSV 36 h plus Toll-scrambled-siRNA group, and WSSV 36 h plus Toll-siRNA group). Expression of *PcALF2* mRNA in intestine after injection with *PcToll5*-siRNA **(B)** or *PcToll6*-siRNA **(E)** *in vivo*. Expression of *PcALF3* in intestine after injection with *PcToll5*-siRNA **(C)** or *PcToll6*-siRNA **(F)**. Error bars indicate standard error and asterisks indicate statistical significance (^∗∗^*P* < 0.01, ^∗^*P* < 0.5).

The expression of *PcToll5* or *PcToll6* in the corresponding siRNA interference group was significantly knocked down in the intestine of *P. clarkii* at 36 h compared with that in the WSSV group. Control siRNA-scrambled did not affect the gene expression, indicating that Toll-siRNA is highly specific to *PcToll5* or *PcToll6*. When *PcToll5* or *PcToll6* expression was inhibited, the *ALF* expression was tested through qRT-PCR analysis. As shown in **Figure 6**, two ALFs (*PcALF2* and *PcALF3*) were significantly upregulated 36 h after the WSSV challenge. However, knocked down of *PcToll5* or *PcToll6* inhibited the transcription of these two ALFs.

### Expression of *PcALF2* and *PcALF3* During WSSV Infection

**FIGURE 7:**
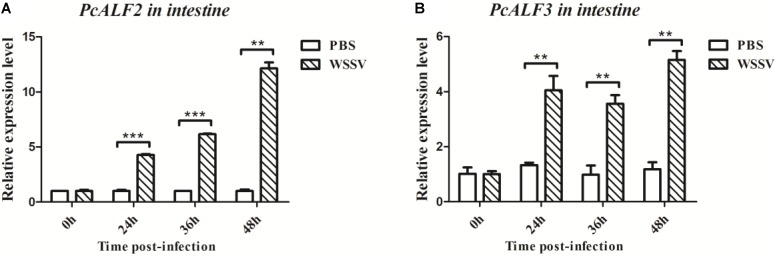
Analysis of *PcALF2* **(A)** and *PcALF3* **(B)** expression in intestine from the crayfish challenged with WSSV using qRT-PCR methods. Asterisks indicate significant differences (^∗∗∗^*P* < 0.001, ^∗∗^*P* < 0.01) compared with values of the control.

*PcALF2* and *PcALF3* genes were characterized, and their expression patterns were determined in the intestine of crayfish to evaluate their roles during WSSV infection. As shown in **Figure 7**, the mRNAs of *PcALF2* and *PcALF3* were upregulated after WSSV infection at several time points (24, 36, and 48 h). By contrast, the expression of *ALFs* did not obviously change after PBS challenge.

## Discussion

Toll receptors are key components in the innate immune responses of invertebrates. Different types of Tolls have been found in several crustacean species (Arts et al., 2007; Mekata et al., 2008; Yang et al., 2008; Lin et al., 2012; Wang et al., 2012, 2015; Yu et al., 2013; Zhou et al., 2015; Lan et al., 2016; Huang et al., 2017). In our previous work, four Toll receptors (*PcToll*, *PcToll2*, *PcToll3*, and *PcToll4*) were identified in the red swamp crayfish *P. clarkii* on the basis of sequence similarities and phylogenetic relationships (Wang et al., 2015; Lan et al., 2016; Huang et al., 2017). In the current study, for the first time, two novel Tolls (*PcToll5* and *PcToll6*) were characterized in *P. clarkii*. Based on the phylogenetic analyses, PcToll5 and PcToll6 exhibited high similarity to crab Toll protein found in *P. trituberculatus* and shrimp Toll proteins in *M. rosenbergii*, *P. clarkii*, and *L. vannamei* and clustered on one branch. PcToll5 and PcToll6 contain more than 20 LRR-related motifs, a transmembrane region, and a TIR domain. LRRs are found in more than 2000 proteins from viruses to eukaryotes (Enkhbayar et al., 2004). Most LRRs are composed of 2–45 motifs, which contain 20–30 amino acids in length and provide a structural framework of protein–protein interactions (Kobe and Kajava, 2001; Enkhbayar et al., 2004). Proteins containing LRRs play a significant role in a number of biological processes, such as signal transduction, DNA repair, cell adhesion, recombination, RNA processing, transcription, disease resistance, apoptosis, and immune response (Rothberg et al., 1990). LRRs are accompanied by cysteine-rich domains: an N-terminal LRR domain and a C-terminal LRR domain. Members of the Toll family are type I transmembrane receptors, which are characterized by an intracellular 200 residue domain with interleukin-1 receptor (IL-1R) and a Toll/IL-1R homologous region (TIR). The TIR domain is essential for Toll proteins and necessary for downstream signal transduction (Kawai and Akira, 2010). This domain is highly conserved not only among different TLRs of one species but also among different animal species (Werling et al., 2009).

*PcToll5* and *PcToll6* exist in different tissues of crayfish. A high expression of *PcToll5* or *PcToll6* was observed in the intestine or gills. The intestine provides an active environment for a variety of microbes, including pathogens, because of its digestion and absorption functions (Huang and Ren, 2015). The gills are frequently exposed to the environment because they are relevant for water and air exchange (Huang X. et al., 2016). The different Tolls in *L. vannamei* were constitutively expressed in all tested tissues (Wang et al., 2012). Tolls identified in three crab species were also widely expressed in few tissues (Lin et al., 2012; Yu et al., 2013; Zhou et al., 2015). Our investigation on the anti-WSSV functions of *PcToll5* and *PcToll6* showed that WSSV upregulated the expression of *PcToll5* and *PcToll6* in the intestine or gills after 24, 36, and 48 h of viral challenge. The *FcToll* expression in the lymphoid organ of *F. chinensis* was downregulated at early periods after WSSV challenge (Yang et al., 2008). Hemolytic *PcToll3* transcription was upregulated 12 h after *V. parahemolyticus* injection or 24 h post WSSV challenge (Lan et al., 2016). WSSV also upregulated the expression of *MrToll* in the gills of *M. rosenbergii* after 24, 36, and 48 h of viral challenge (Feng et al., 2016). Another *MrToll* was enhanced 3–12 h after *Aeromonas caviae* stimulation and decreased to basal levels at 24 h post challenge (Srisuk et al., 2014).

Increasing lines of evidence have indicated that the crustacean Toll signaling pathway exerts anti-bacterial, anti-fungal, and anti-viral functions by regulating the expression of immune-related genes (Wang et al., 2015; Lan et al., 2016; Feng et al., 2016). AMPs are one of the major constituents of the invertebrate innate immune system and function as the front line of host defense against microbial infection (Silva et al., 2013). Crustacean AMPs include several families such as Crus, ALFs, and Lys (Lin et al., 2015). Most research focused on the anti-bacterial function of ALFs, which were reported to be involved in anti-viral defense. *Pacifastacus leniusculus* ALF interfered with WSSV replication and effectively protected crayfish from WSSV infection (Liu et al., 2006). *P. monodon* ALFPm3 exhibited anti-WSSV activity by interacting with the envelope protein WSSV189 and other WSSV structural proteins (Tharntada et al., 2009; Suraprasit et al., 2014). nLvALF1 with its SNP polymorphisms participated in defense against WSSV in *L. vannamei* (Liu et al., 2014). In the present study, when *PcToll5* or *PcToll6* was silenced, the expression levels of *PcALF2* and *PcALF3* were significantly suppressed after WSSV challenge; hence, *PcALF2* and *PcALF3* might be regulated by the Toll-mediated signaling pathway. In *P. clarkii*, *PcToll* was involved in regulation of *Cru1*, *Cru2*, *ALF2*, and *Lys1* expression (Wang et al., 2015). *PcToll3* silencing influenced the expression of *Cru1* and *Lys1* after *Vibrio* challenge (Lan et al., 2016). In the giant freshwater prawn *M. rosenbergii*, *ALF2*, *ALF3*, *ALF4*, and *ALF5* were regulated by *MrToll* in the gills during WSSV challenge (Feng et al., 2016). In the present work, overexpression of PcToll5 or PcToll6 in *Drosophila* S2 cells was conducted to determine the roles of PcToll5 and PcToll6 in AMP expression. The results also implied that PcToll5 and PcToll6 activated the transcription of AMPs such as *AttA*, *Mtk*, *Drs*, and *Pen4*. Based on RNAi and over-expression assay, PcToll5 and PcToll6 played important roles in anti-WSSV immune defense by regulating ALF gene expression.

## Conclusion

Two novel Tolls (*PcToll5* and *PcToll6*) from *P. clarkii* were cloned and characterized. *PcToll5* and *PcToll6* were broadly expressed in all tested tissues of crayfish, and their transcription was induced by WSSV challenge. Overexpression and RNAi experiments showed that PcToll5 and PcToll6 could regulate the expression of AMPs. All these results suggest that *PcToll5* and *PcToll6* may participate in innate immunity against pathogen infection. However, further studies are required to clarify the specific function and immune mechanism of *PcTolls* in crayfish immune defense.

## Ethics Statement

We declare that appropriate ethical approval and licenses were obtained during our research.

## Author Contributions

YH and YC carried out the experiments, contributed reagents and materials. YH, KH, and QR designed the experiments and analyzed the data. YH and QR wrote the manuscript. All authors gave final approval for publication.

## Conflict of Interest Statement

The authors declare that the research was conducted in the absence of any commercial or financial relationships that could be construed as a potential conflict of interest.

## Footnotes

1 https://blast.ncbi.nlm.nih.gov/Blast.cgi

2 http://www.expasy.ch/

3 http://smart.embl-heidelberg.de/
