# Supplementary material for: Cloning and Characterization of Two Toll Receptors (PcToll5 and PcToll6) in Response to White Spot Syndrome Virus in the Red Swamp Crayfish Procambarus clarkii
Source: Front Physiol. 2018 Jul 16;9:936. doi: 10.3389/fphys.2018.00936 (PMC6060793; doi:10.3389/fphys.2018.00936)
Supplement: Supplementary file 1 [file Data_Sheet_1.doc]

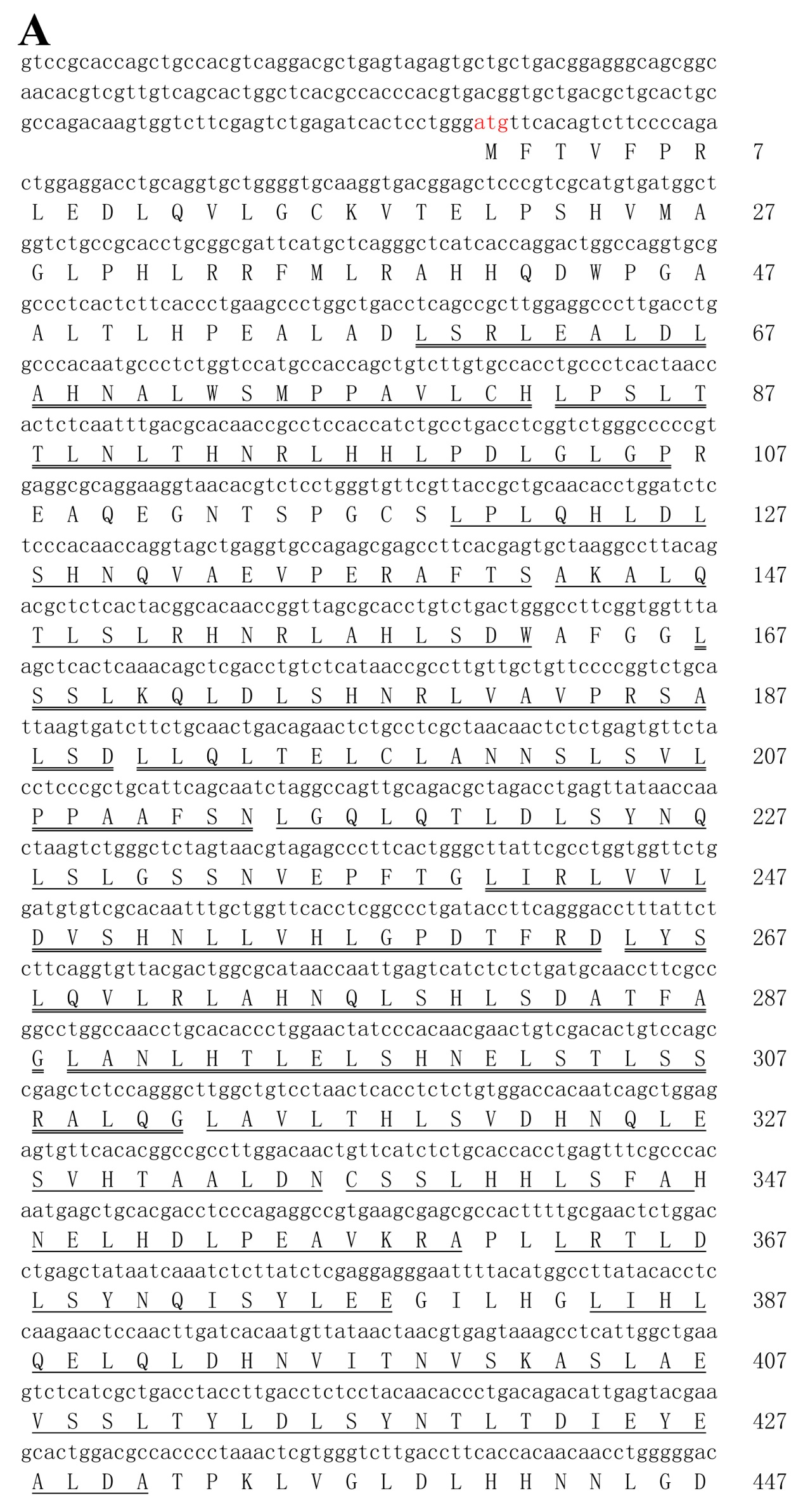


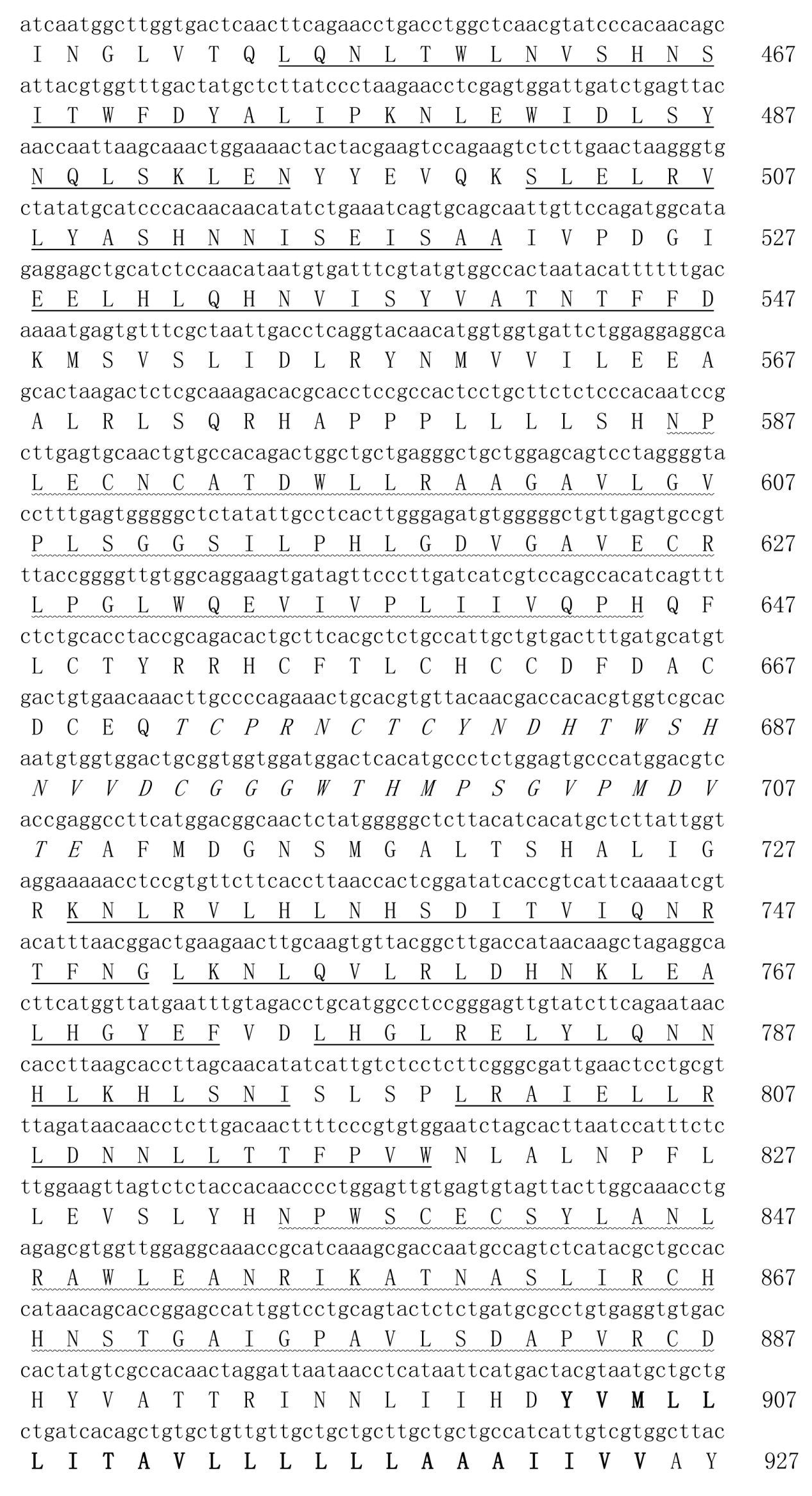


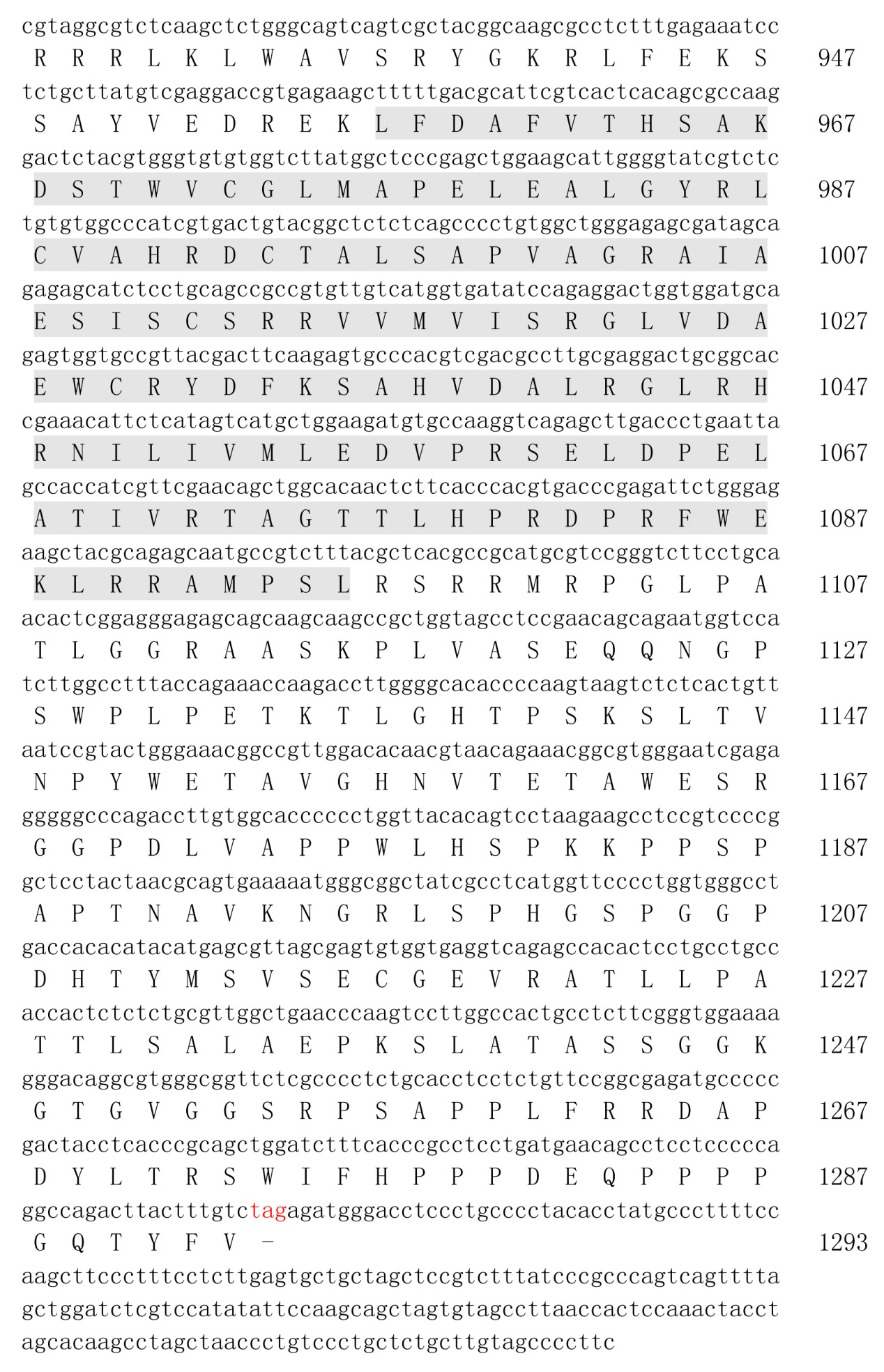


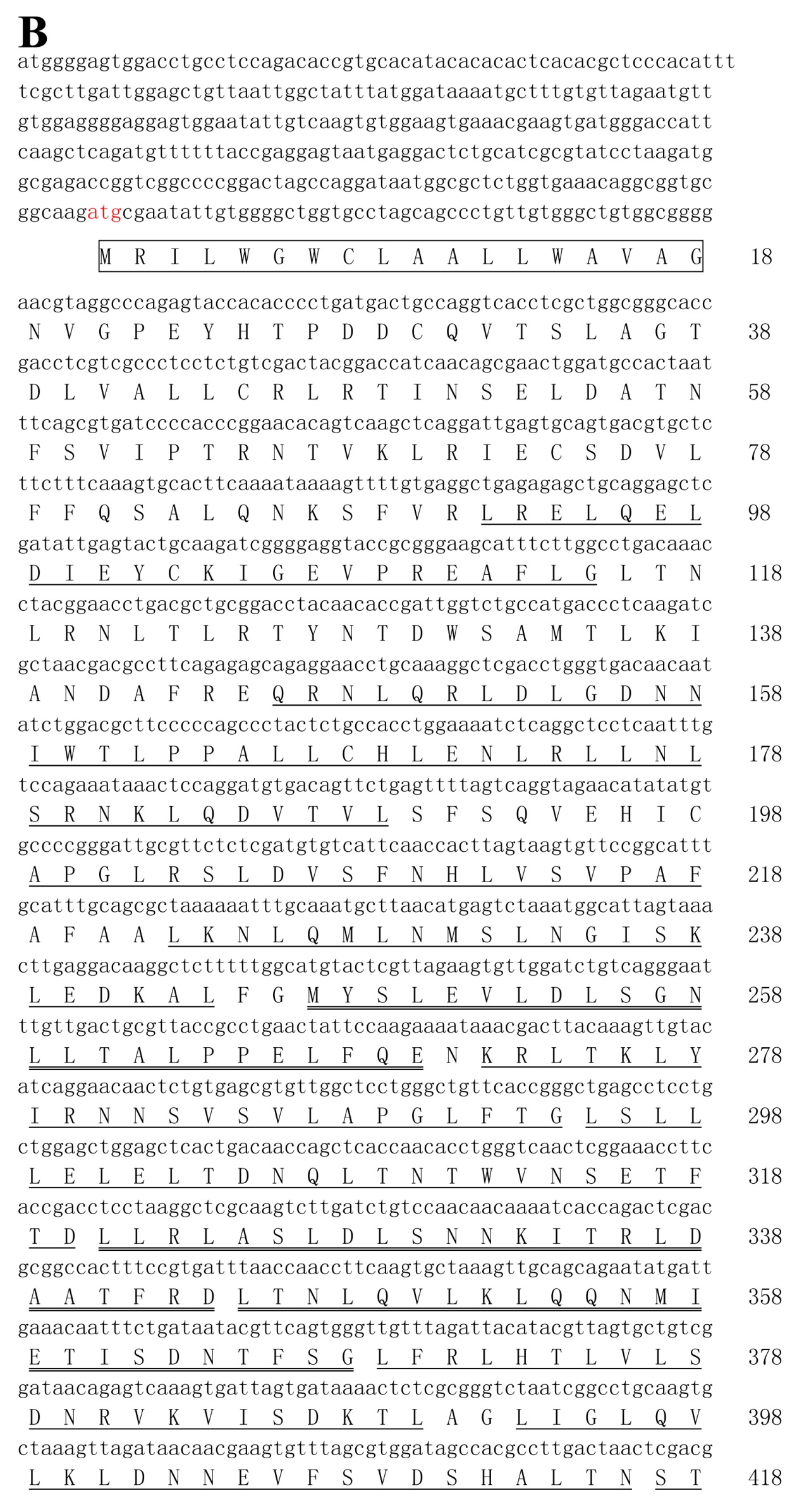


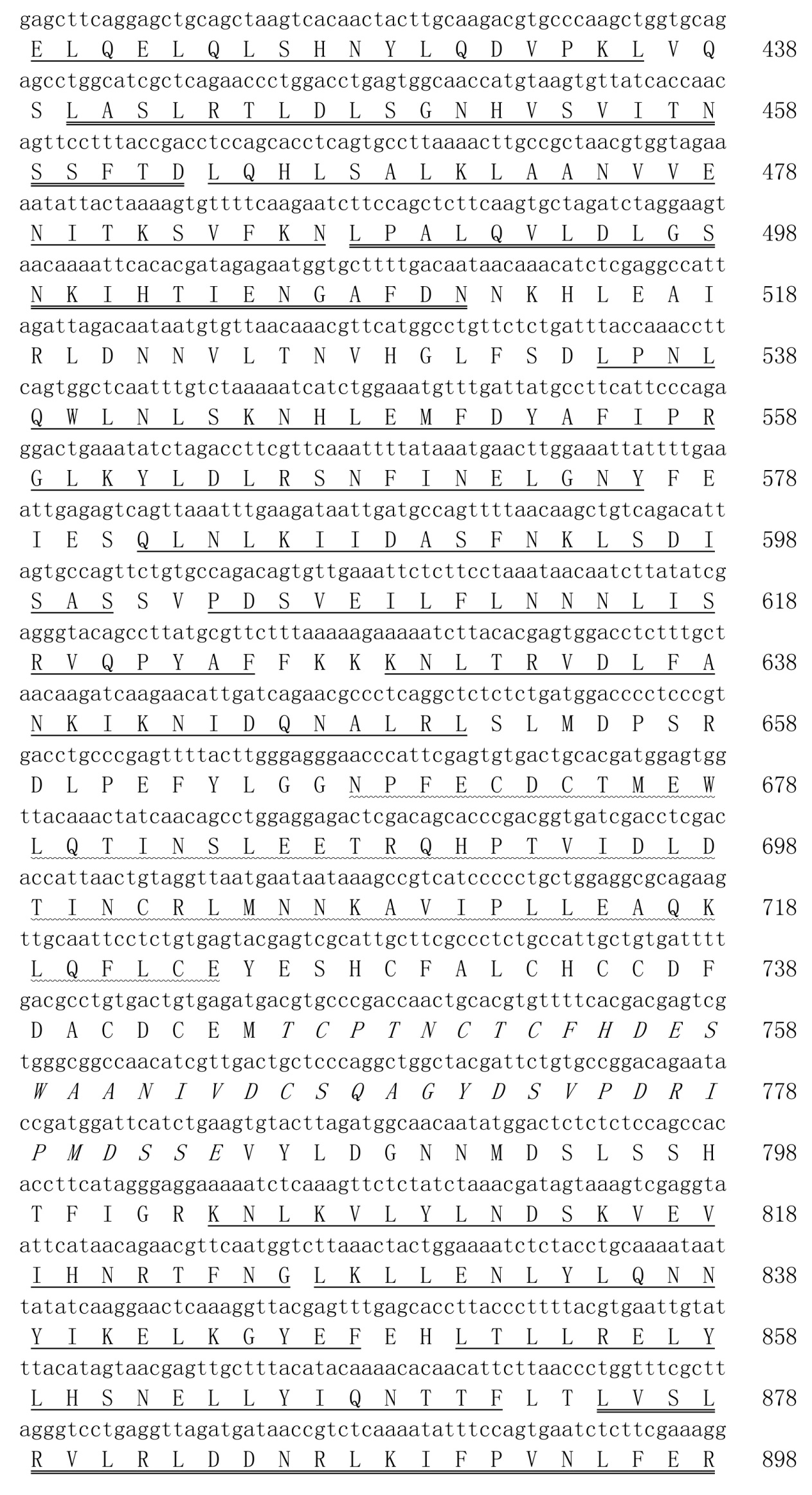


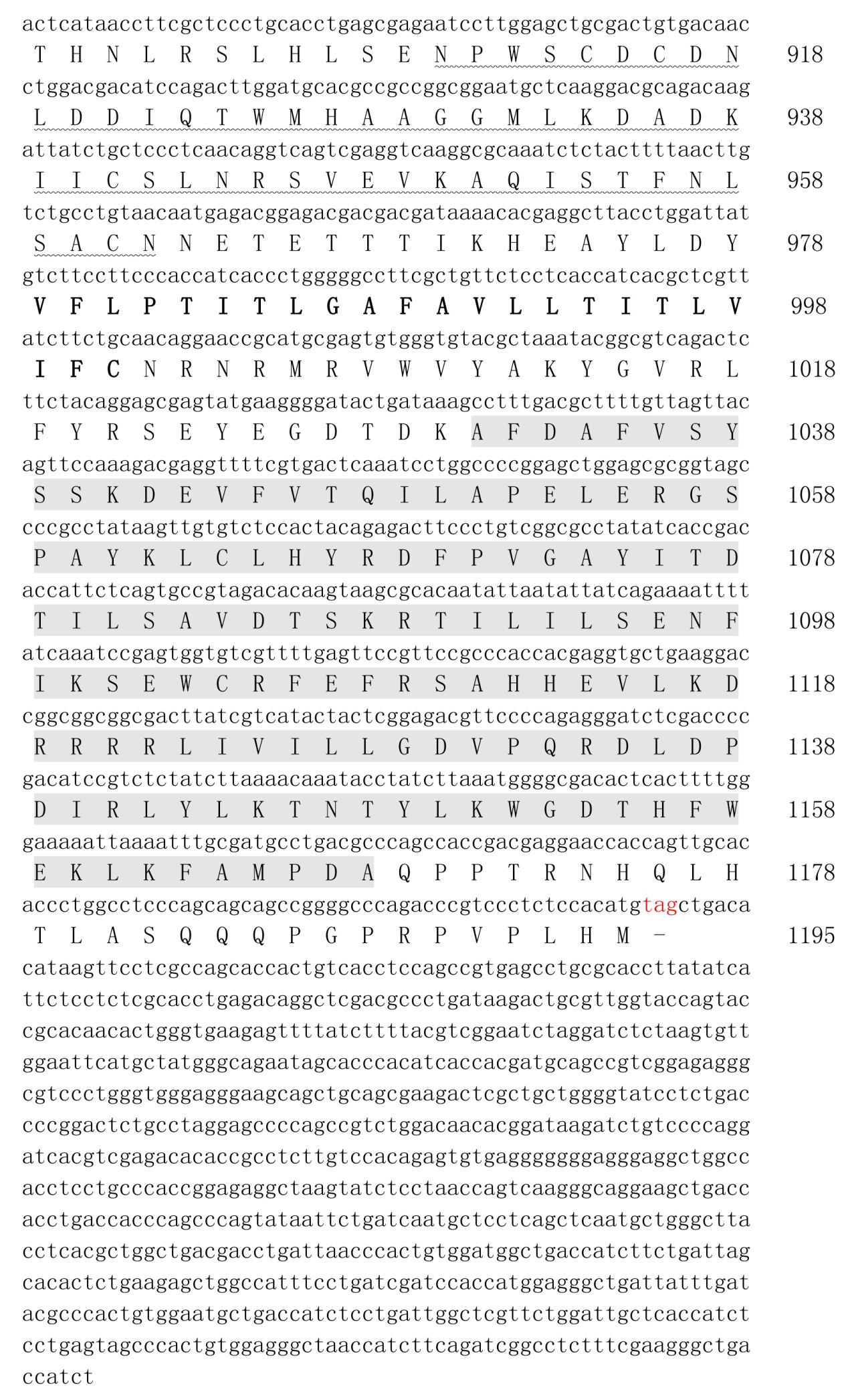


**Fig. S1.** Nucleotides and deduced amino acid sequences of PcToll5 (A) and PcToll6 (B) from *P. clarkii*. Start codon (ATG) and stop codon (TAG) are shown in red. Signal peptide sequences are boxed. The LRR domains are underlined, and the LRR TYP domains are double underlined. The wavy line represents the LRR CT regions. The LRR NT is labeled in italics. Transmembrane region is shown in bold, and shaded sequences denote the TIR domain.


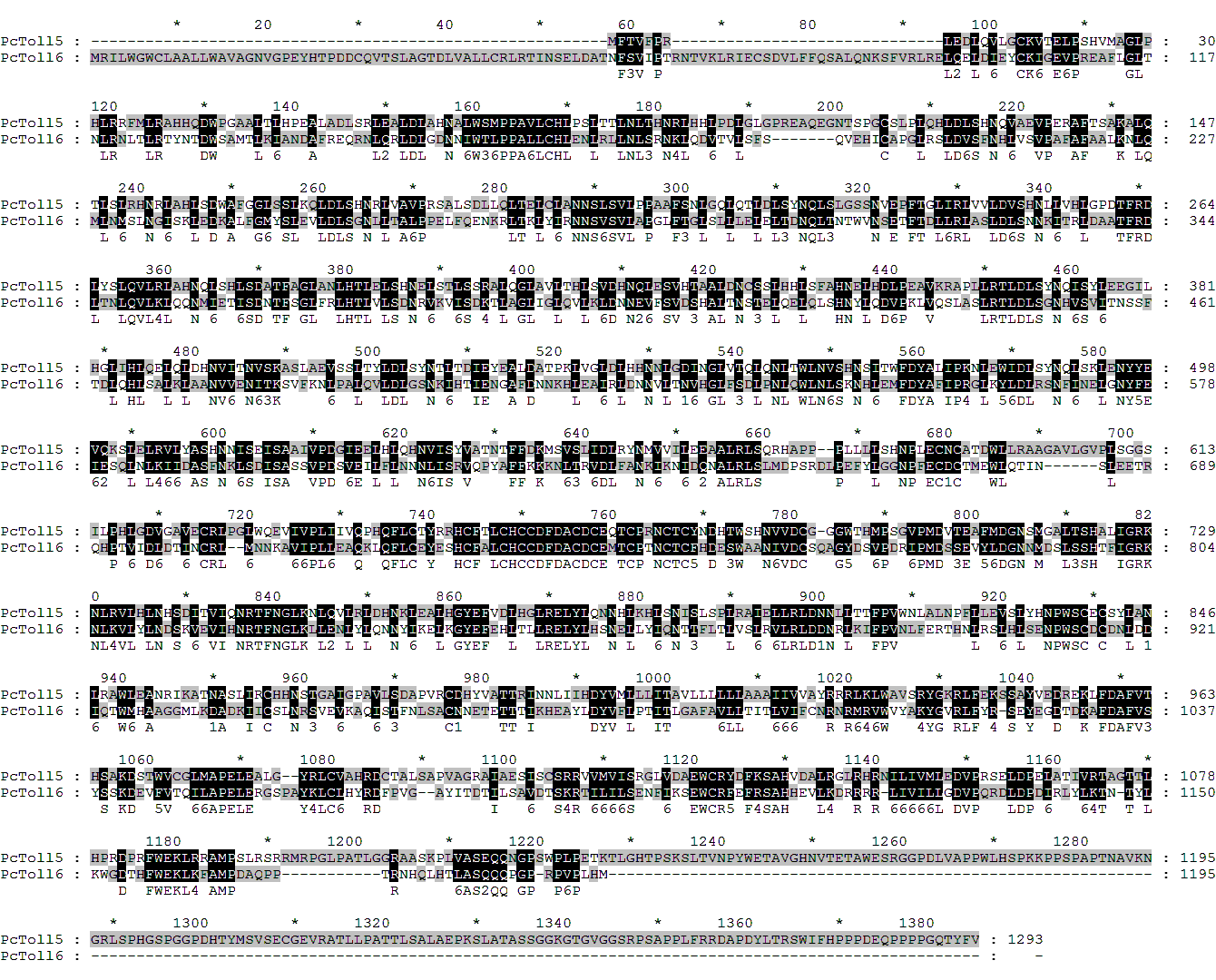


**Fig. S2.** Multiple sequence alignment by GENEDOC among PcToll5 and PcToll6 from *P. clarkii* according to the deduced amino acid sequence.
